# Virological outcomes and treatment retention in North Vietnam amidst transition to social insurance-based HIV services and dolutegravir-based regimens

**DOI:** 10.1038/s41598-025-26866-5

**Published:** 2025-11-28

**Authors:** Shoko Matsumoto, Moeko Nagai, Linh Khanh Tran, Tsunefusa Hayashida, Hoai Dung Thi Nguyen, Truong Manh Nguyen, Giang Van Tran, Daisuke Mizushima, Junko Tanuma, Kinh Van Nguyen, Thach Ngoc Pham, Shinichi Oka

**Affiliations:** 1 AIDS Clinical Center, National Center for Global Health and Medicine, Japan Institute for Health Security, Tokyo, Japan; 2 National Hospital for Tropical Diseases, Hanoi, Vietnam; 3 Department of Infectious Diseases, Hanoi Medical University, Hanoi, Vietnam; 4 1-21-1, Toyama, Shinjuku-ku, Tokyo, 162-8655 Japan; 5 Department of Infectious Diseases, School of Medicine, International University of Health and Welfare, Chiba, Japan; 6 Department of Global Health and Infectious Diseases, Graduate School of Public Health, International University of Health and Welfare, Tokyo, Japan

**Keywords:** HIV/AIDS, Drug resistance, Vietnam, Dolutegravir, Social health insurance, HIV infections, Epidemiology

## Abstract

Vietnam has been experiencing the transition from donor-based to social insurance-based antiretroviral therapy (ART), the COVID-19 pandemic, and expansion of dolutegravir (DTG) use. We assessed virological outcomes, care retention, and effectiveness and tolerability of switching to DTG-containing regimen among people living with HIV (PLHIV) during these changes. PLHIV with suppressed HIV viral load (HIV-VL) who were receiving ART at 11 facilities in North Vietnam were enrolled in a prospective cohort from December 2019 through September 2021 and followed up until March 2023. This cohort of 2,233 PLHIV on ART maintained viral suppression rates (HIV-VL < 50 copies/mL) of > 90% and care retention rates of > 87% throughout the study period. Incidence of viremia (HIV-VL ≥ 200 copies/mL) was 3.2/100 person-years during follow-up. Only 32 (1.4%) PLHIV had any drug resistance mutations; no DTG-associated mutation was observed. Of 1,891 who switched to DTG-containing regimens, 292 (15.4%) discontinued DTG, most commonly owing to DTG stockout (80.8%). Average weight gain was greater in the first measurement after switching than in subsequent measurements. In conclusion, successful maintenance of virologic outcomes of ART and high treatment retention were observed amid various social and clinical changes in Vietnam. These real-world data support the national rollout of DTG.

**Supplementary Information:**

The online version contains supplementary material available at 10.1038/s41598-025-26866-5.

## Introduction

 Vietnam has continued to make progress toward ending the HIV epidemic. By 2022, more than 70% of the estimated 250,000 people living with HIV (PLHIV) have received antiretroviral therapy (ART), and the number of new infections has decreased by approximately 56% since 2010^1^. Furthermore, a nationally representative survey of PLHIV on ART conducted during 2020 in Vietnam reported a viral suppression rate of 96.4% at 12 ± 3 months after ART initiation^2^, which meets the third 95% target set by the Joint United Nations Programme on HIV/AIDS^3^. However, in recent years, Vietnam has been facing formidable challenges in sustaining these gains.

The first challenge concerns HIV/AIDS funding. Historically, the HIV/AIDS response in Vietnam has mainly been financed by international donors such as the Global Fund. However, since 2016, international partners have begun gradually shifting their support from financial aid to technical assistance as Vietnam’s economy grows. To fill the funding gap in sustaining the effectiveness of HIV/AIDS programs, the Vietnamese government has committed to integrating HIV services into the social health insurance (SHI) scheme^4–6^. In this context, domestic resources, which supported 30% of the HIV/AIDS response in 2015, have increased by approximately 3.5% annually, reaching 49% in 2022^7,8^. However, this transition may impose a series of important changes on beneficiaries and providers. In particular, decentralization of HIV services and out-of-pocket costs can pose barriers to continuing ART for PLHIV. For example, according to the initial regulations, PLHIV could only receive SHI-covered HIV services at neighborhood hospitals where they were registered. However, PLHIV tend to avoid visiting their registered hospitals owing to fear of stigma in their community. This might increase disengagement from ART and the incidence of virological failure and HIV drug resistance (HIV-DR).

Funding challenges with respect to the HIV/AIDS response could be further exacerbated by the COVID-19 pandemic, which has infected more than 11 million people in Vietnam as of September 2023^9^. PLHIV might be affected by COVID-19 in various ways, including having an elevated risk of mortality^10,11^, interruption of HIV treatment and care^12–15^ owing to a stagnated ART drug supply^16^ and traffic restrictions, and deterioration of mental health^17^. These may lead to poor treatment outcomes.

While facing these challenges, a clinical change has also taken place that could be key to the success of ART. A novel integrase strand transfer inhibitor (INSTI), dolutegravir (DTG), was introduced in Vietnam in 2019. DTG reportedly has advantages in terms of virological efficacy^18,19^, a high genetic barrier to drug resistance^20,21^, low risk of adverse reactions^22^ and drug–drug interactions^20^, and once-daily dosing schedule. Despite such advantages, several studies have reported weight gain in patients switching to a DTG-containing regimen^23,24^. The World Health Organization (WHO) recommends DTG-containing regimens as the preferred first- and second-line ART regimens for PLHIV^25,26^. Following the WHO guidelines, previously used non-nucleoside reverse transcriptase inhibitor (NNRTI)-containing regimens, mainly tenofovir/lamivudine/efavirenz (TDF/3TC/EFV), have been replaced by DTG-containing regimens (mainly generic TDF/3TC/DTG) in many ART clinics of Vietnam. However, effectiveness, tolerability, and weight increase after switching to DTG-containing regimens have been mostly reported from Western and African countries, and real-world reports from Asia, including Vietnam, are lacking.

Amid the above-mentioned changes in Vietnam, we aimed to assess virological outcomes, including acquired drug resistance, retention in care, and effectiveness and tolerability of switching to DTG-containing regimens, using data collected over the past 3 years from PLHIV in North Vietnam. The changes that have been taking place in Vietnam are also being experienced in other developing countries. The results of the present study will aid in clarifying achievements and challenges regarding recent clinical and policy changes to help Vietnam and other countries to develop better strategies for HIV/AIDS control.

## Methods

### Study setting and population

This was an analysis of prospectively collected data from a multicenter observational cohort of PLHIV on ART (SATREPS-HIV). This cohort was established under a Japanese government program, the “Science and Technology Research Partnership for Sustainable Development (SATREPS),” with the primary objective of assessing changes in treatment outcomes with the transition to SHI-based HIV services in Vietnam. One national-level hospital (National Hospital for Tropical Diseases, NHTD) and ten local ART facilities (seven provincial/city-level and three district-level facilities) in North Vietnam were included in the SATREPS-HIV (Supplementary Table S1). These ten local facilities comprised Dong Da General Hospital (DDGH), 09 Hospital, Nam Tu Liem Health Center (NTL), Quang Ninh General Hospital (QNGH), Hung Yen Hospital of Tropical Diseases (HYTD), Hai Duong Hospital for Tropical Diseases (HDHTD), Thanh Son District Medical Center (TSMC), Yen Binh District Medical Center (YBMC), Nghe An General Hospital (NAGH), and Ha Tinh Center for Disease Control and Prevention (HTCDC). These facilities were selected in consultation with the Vietnamese Ministry of Health from multiple perspectives, including region, facility level, HIV prevalence, and support from overseas donors. Additionally, because SATREPS was implemented within the framework of the Japanese government’s official development assistance, some facilities were selected with an intention to provide technical assistance to facilities with insufficient access to HIV services, such as HIV viral load (HIV-VL) testing.

The inclusion criteria for this study were as follows: written consent given to participate in the study, age 16 years or older, Vietnamese nationality, confirmed HIV infection, receiving ART for more than 6 months, holding an SHI card, and under the lower limit (20 or 50 copies/mL) for the most recent available HIV-VL. Owing to budget constraints, we principally randomly selected 100 participants from each local facility (total of 1000 participants from ten local facilities) for enrollment. However, a few facilities (TSMC and YBMC) only had approximately 100 HIV-infected outpatients. In these cases, all patients who met the inclusion criteria were enrolled. Regarding patient recruitment at NHTD, prior to SATREPS-HIV, the National Center for Global Health and Medicine established a hospital-based cohort of PLHIV taking ART to monitor clinical outcomes at NHTD. In this cohort, 1,287 PLHIV were under followed-up as of October 2019. Therefore, for convenience, we included all patients who were participating in this cohort and met the above inclusion criteria.

Patient enrollment was conducted during two periods: from December 2019 to March 2020 at NHTD and four local facilities, and from April to September 2021 at six other local facilities (Supplementary Table S1). This was because at the beginning of the SATREPS-HIV, it was unclear how the transition to SHI-based HIV services would proceed in many facilities. Therefore, six facilities were selected while monitoring the transition progress. If the selected patients did not appear at study sites for enrollment or did not give written informed consent, study participants were randomly selected until 100 participants were enrolled.

Participants were followed up at their registered facilities every 6 months to have a blood sample collected for HIV-VL and HIV-DR testing during their regular consultations. HIV-DR testing was performed for those with an HIV-VL ≥ 1,000 copies/mL. Each facility followed two study visit cycles during the Japanese fiscal year (FY), beginning in April and ending in March of the following year. The first visit cycle fell between April to September, and the second cycle fell between October to March. However, the duration of each cycle was extended during the period when transportation was restricted owing to the COVID-19 pandemic. Nevertheless, study visits were missed by those who were unable to visit the study site during the study period or who received ART by mail or at other facilities. All data collected in SATREPS-HIV, including demographics (e.g., sex, age) and HIV-related information (e.g., route of HIV transmission, ART history), clinical information (e.g., ART history, weight, HIV-VL and HIV-DR results), and follow-up status were reported by physicians or nurses at each study site using a cloud database. As for weight, only data from NHTD were collected because a weight scale was unavailable at many other hospitals.

### Outcomes

The virological outcomes of ART were assessed using HIV-VL and acquired drug resistance detected during the follow-up period. Plasma samples were collected at each study site and were transported to NHTD for HIV-VL and HIV-DR testing. The quantitative measurement of HIV-VL was carried out using two automated systems, COBAS AmpliPrep/COBAS TaqMan and cobas 6800 (Roche Diagnostics, Basel, Switzerland) in the NHTD laboratory. The results for HIV-VL were divided into four categories: <50 copies/mL, 50–199 copies/mL, 200–999 copies/mL, and ≥ 1,000 copies/mL. We examined the risk of viremia, defined as HIV-VL ≥ 200 copies/mL^27,28^, and its associated factors. HIV-DR genotyping was performed for study participants with treatment failure (HIV-VL ≥ 1,000 copies/mL). Sanger sequencing was conducted using the 3500 Genetic Analyzer (Thermo Fisher Scientific, Waltham, MA, USA) for the regions HIV-1 protease (amino acids 1–99), reverse transcriptase (1–560), and integrase (1–289) using the in-house method^29^. The Stanford HIV Drug Resistance Database (https://hivdb.stanford.edu/) was used to evaluate drug resistance mutations (DRMs) to nucleos(t)ide reverse transcriptase inhibitors (NRTIs), NNRTIs, protease inhibitors (PIs), and INSTIs.

Retention in care was assessed according to the follow-up status at each study visit. The status was coded using the following five categories: under follow-up, transfer, death, skip, and loss to follow-up. Skip was defined as one missed appointment, and loss to follow-up was defined as two consecutive missed appointments.

To assess the effectiveness of switching to DTG-containing regimens, we assessed DTG use, viremia, and their associations. Furthermore, to evaluate the tolerability of DTG-containing regimens, the reasons for discontinuation of the regimens were identified and classified into the following categories: lack of effectiveness (either virological, immunological or clinical), toxicity (i.e., renal, allergic, gastrointestinal, neuropsychiatric, hepatic, and osteoarticular dysfunction or reactions potentially owing to the prescribed drug), stockout (defined as a situation in which physicians are unable to prescribe the drug because it is out of stock), and other reasons (e.g., drug–drug interactions, pregnancy or desire to become pregnant, and patient’s or physician’s choice).

To examine weight gain after switching to a DTG-containing regimen, we assessed the average weight per visit among patients from NHTD taking the regimen.

### Statistical analyses

The primary outcomes of this study were virological outcomes (HIV-VL level and acquired DRMs) and treatment retention during the follow-up period. We descriptively analyzed the prevalence of each HIV-VL level and follow-up status according to study visit, acquired DRMs (any DRM and DRMs by drug class) in patients with HIV-VL ≥ 1,000 copies/mL. Additionally, we calculated the incidence of viremia (HIV-VL ≥ 200 copies/mL) during the follow-up period in all study participants. We counted viremia developed at any time after enrollment. The follow-up time was calculated from the first day of clinical follow-up in this study up to the last clinical follow-up or the date of first viremia. Cox proportional hazard models were used to identify factors independently associated with the risk of viremia during the follow-up period. The following covariates were included in the models: facility level, sex, age, injection drug use (IDU), men who have sex with men (MSM), time from HIV diagnosis to registration (< 10 or ≥ 10 years), time from HIV diagnosis to ART initiation (< 1 month, ≥ 1 and < 6 months, ≥ 6 and < 12 months, or ≥ 12 months), the most recent CD4 count before the first visit (< 200/µL, 200–349/µL, or ≥ 350/µL), and HIV-VL at the first visit (< 20/mL, 20–199/mL, 200–999/mL, or ≥ 1000/mL). These covariates were collected at enrollment. Factors that were associated with viremia with a *p*-value < 0.1 in the univariable model were included in the multivariable model.

The secondary outcomes were effectiveness and tolerability of switching to DTG-containing regimens. We descriptively assessed the reasons for discontinuation of DTG-containing regimens. The incidence of viremia after DTG initiation among study participants who used a DTG-containing regimen was also calculated. In this analysis, we only included participants who were on a DTG-containing regimen for at least 12 weeks to ensure sufficient and stable therapeutic effects of DTG, and those who had an HIV-VL test result ≥ 12 weeks after starting DTG. We counted viremia developed after DTG initiation. However, because only participants who had taken DTG-containing regimens for 12 weeks or more were included in this analysis, viremia that developed within 12 weeks after DTG initiation was not counted. The follow-up time was calculated from the first day of DTG initiation to the last clinical follow-up or the date of first viremia. For study participants who discontinued DTG-containing regimens, only the period during which they were taking DTG was included in the follow-up period, and only viremia while taking a DTG-containing regimen was counted. As for examination of weight gain after switching to a DTG-containing regimen, we calculated the difference in mean weights among four visits by sex, from October 2019 to February 2023, and a paired *t*-test was conducted. Only patients from NHTD who had data for all visits were included in the examination. The analyses were performed using SAS 9.4 software (SAS Institute Inc., Cary, NC, USA) or Stata 16 (StataCorp, College Station, TX, USA). All tests were two-sided, with the significance level set at 5%. Missing data were excluded from the analyses.

## Results

Among a total of 7,432 patients who met the inclusion criteria, 2,233 were included in this study (Table 1). Approximately half of the included patients were from NHTD (*n* = 1,228, 55.0%); the other half were from provincial-level (30.5%) or district-level (14.6%) facilities. In two facilities (NAGH and HTCDC), the volume of blood samples from two patients was insufficient for HIV-VL testing at enrollment. These patients were unable to return to the facility for retesting during the first study period; therefore, two additional patients were selected so as to reach 100, resulting in a total of 102 enrolled patients.

### Virological outcomes of ART

**Fig. 1:**
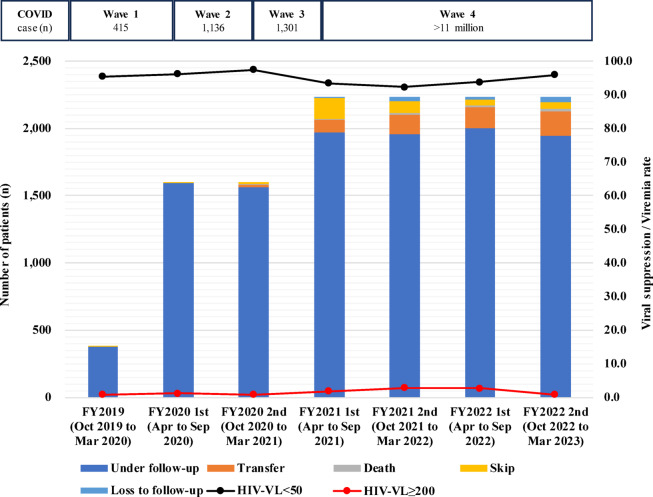
HIV viral load and follow-up status. The Japanese fiscal year (FY) starts in April and ends in March of the following year. Each study site followed two study visit cycles during the Japanese fiscal year. The first visit cycle fell between April to September, and the second cycle fell between October to March. In FY 2019, all data were collected in December 2019. NHTD and four local facilities (DDGH, 09 Hospital, NTL, and QNGH) started data collection between December 2019 and March 2020. The other six local facilities (HYTD, HDHTD, TSMC, YBMC, NAGH, and HTCDC) started data collection between April and September 2021. Skip is defined as one missed appointment. Loss to follow-up is defined as two consecutive missed appointment. COVID-19 wave was based on NT Trang et al.^54^. HIV-VL, HIV viral load; NHTD, National Hospital for Tropical Diseases; DDGH, Dong Da General Hospital; NTL, Nam Tu Liem Health Center; QNGH, Quang Ninh General Hospital; HYTD, Hung Yen Hospital of Tropical Diseases; HDHTD, Hai Duong Hospital for Tropical Diseases; TSMC, Thanh Son District Medical Center; YBMC, Yen Binh District Medical Center; NAGH, Nghe An General Hospital; HTCDC, Ha Tinh Center for Disease Control and Prevention.

Figure 1 shows the HIV-VL and follow-up status between FY 2019 and FY 2022. Viral suppression rates (HIV-VL < 50 copies/mL) were maintained at 90% or higher throughout the study period and were just below 95% (but > 90%) at only three study visits in FY 2021 and FY 2022. Rates of viremia (HIV-VL ≥ 200 copies/mL) remained less than 3.0%. During the follow-up period, 75 of 2,233 participants (3.4%) had HIV-VL > 1000 copies/mL, and sequence data were successfully obtained from 69 participants for HIV-DR testing. In total, 32 (1.4%) had any DRMs. NNRTI-associated DRMs were the most common (1.3%), followed by NRTI-associated DRMs (0.8%). PI-associated DRMs were less common (0.1%), and no INSTI-associated DRMs was observed (Table 2). Supplementary Table S2 provides details of DRMs according to drug class. M184V and K103N were the most common DRMs, which are highly resistant to available antiretrovirals in Vietnam (to 3TC, and to nevirapine and EFV, respectively).

**Table 1:** Characteristics of study participants.

Variables	*n* (%)	Variables	*n* (%)
All	2,233 (100.0)	Time from HIV diagnosis to ART initiation	
Facility		Median (IQR), months	1.0 (0.0, 12.0)
NHTD	1,228 (55.0)	<1	792 (35.5)
DDGH	90 (4.0)	≥1, <6	661 (29.6)
09 Hospital	88 (3.9)	≥6, < 12	178 (8.0)
NTL	98 (4.4)	≥12	587 (26.3)
QNGH	99 (4.4)	NA	15 (0.7)
HYTD	99 (4.4)	Route of HIV transmission (multiple choice)	
HDHTD	100 (4.5)	IDU	595 (26.7)
TSMC	128 (5.7)	MSM	60 (2.7)
YBMC	99 (4.4)	Other sexual contacts	1,440 (64.5)
NAGH	102 (4.6)	Unknown	174 (7.8)
HTCDC	102 (4.6)	ART regimen at the first visit	
Facility level		TDF/3TC/DTG	1,280 (57.3)
National	1,228 (55.0)	TDF/3TC/EFV600	339 (15.2)
Provincial	680 (30.5)	TDF/3TC/EFV400	309 (13.8)
District	325 (14.6)	AZT/3TC/NVP	111 (5.0)
Sex		TDF/3TC/LPV/r	61 (2.7)
Male	1,311 (58.7)	AZT/3TC/EFV600	59 (2.6)
Female	922 (41.3)	Other	73 (3.4)
Age		CD4 (/mL) before the first visit	
Median (IQR)	41 (37, 46)	Median (IQR)	523 (398, 683)
<30	131 (5.9)	<200	48 (2.2)
30–39	758 (34.0)	200–349	210 (9.4)
40–49	1,036 (46.4)	≥350	1,225 (54.9)
≥50	308 (13.8)	NA	750 (33.6)
Time from HIV diagnosis to the first visit		HIV-VL (copies/mL) at the first visit	
Median (IQR), years	9.3 (6.8, 12.5)	Median (IQR)	TND (TND, < 20)
<10	1,274 (57.1)	<20	1,970 (88.2)
≥10	953 (42.7)	20–199	236 (10.6)
NA	6 (0.3)	200–999	11 (0.5)
		≥1000	16 (0.7)
		NA	0 (0.0)

IQR, interquartile range; ART, antiretroviral therapy; IDU, injection drug use; MSM, men who have sex with men; HIV-VL, HIV viral load; NA, not available; NHTD, National Hospital for Tropical Diseases; DDGH, Dong Da General Hospital; NTL, Nam Tu Liem Health Center; QNGH, Quang Ninh General Hospital; HYTD, Hung Yen Hospital of Tropical Diseases; HDHTD, Hai Duong Hospital for Tropical Diseases; TSMC, Thanh Son District Medical Center; YBMC, Yen Binh District Medical Center; NAGH, Nghe An General Hospital; HTCDC, Ha Tinh Center for Disease Control and Prevention.

**Table 2:** Prevalence of drug resistance mutations.

	*n* (%)^a^
Participants who had HIV-VL ≥ 1000 copies/mL at and after the first visit	75 (3.4)
Participants with HIV-DR testing completed (any region) at least once	69 (3.1)
DRMs (any)	32 (1.4)
DRMs (any NRTIs)	17 (0.8)
DRMs (any NNRTIs)	30 (1.3)
DRMs (any PIs)	3 (0.1)
DRMs (any INSTIs)^b^	0 (0.0)
DRMs (NRTIs + NNRTIs)	16 (0.7)
DRMs (NRTIs + PIs)	1 (0.0)
DRMs (NRTIs + NNRTIs + PIs)	1 (0.0)

^a^ The percentage was calculated with the total number of participants as the denominator (*n* = 2,233).

^b^ Drug resistance testing covering the integrase region was performed in patients who had received or were receiving a DTG-containing regimen (*n* = 39).

HIV-VL, HIV viral load; HIV-DR, HIV drug resistance; DRMs, drug resistance mutations; NRTIs, nucleos(t)ide analogue reverse transcriptase inhibitors; NNRTIs, non-nucleoside reverse transcriptase inhibitors; PIs, protease inhibitors; INSTIs, integrase strand transfer inhibitors.

Sanger sequencing was conducted for the regions of HIV-1 protease (amino acids 1–99), reverse transcriptase (1–560), and integrase (1–289).

### Retention in care

High rates of retention in care were observed, with follow-up rates ranging from 87.2% to 99.7% during the follow-up period. Loss to follow-up was rare, with the highest rate (1.6%) at the final study visit in FY 2022. The number of transfers increased gradually from FY 2021, reaching 8.1% at the final study visit in FY 2022 (Fig. 1).

### Incidence of viremia and its associated factors

A total of 144 participants (6.4%) experienced viremia (HIV-VL ≥ 200 copies/mL) during the follow-up period, with an incidence of 3.2/100 person-years. In Cox regression analysis, the predictors of viremia in both univariable and multivariable models were facility level, CD4 count before the first visit, and HIV-VL at the first visit (Supplementary Table S3).

### Effectiveness and tolerability of switching to DTG-containing regimens

**Fig. 2:**
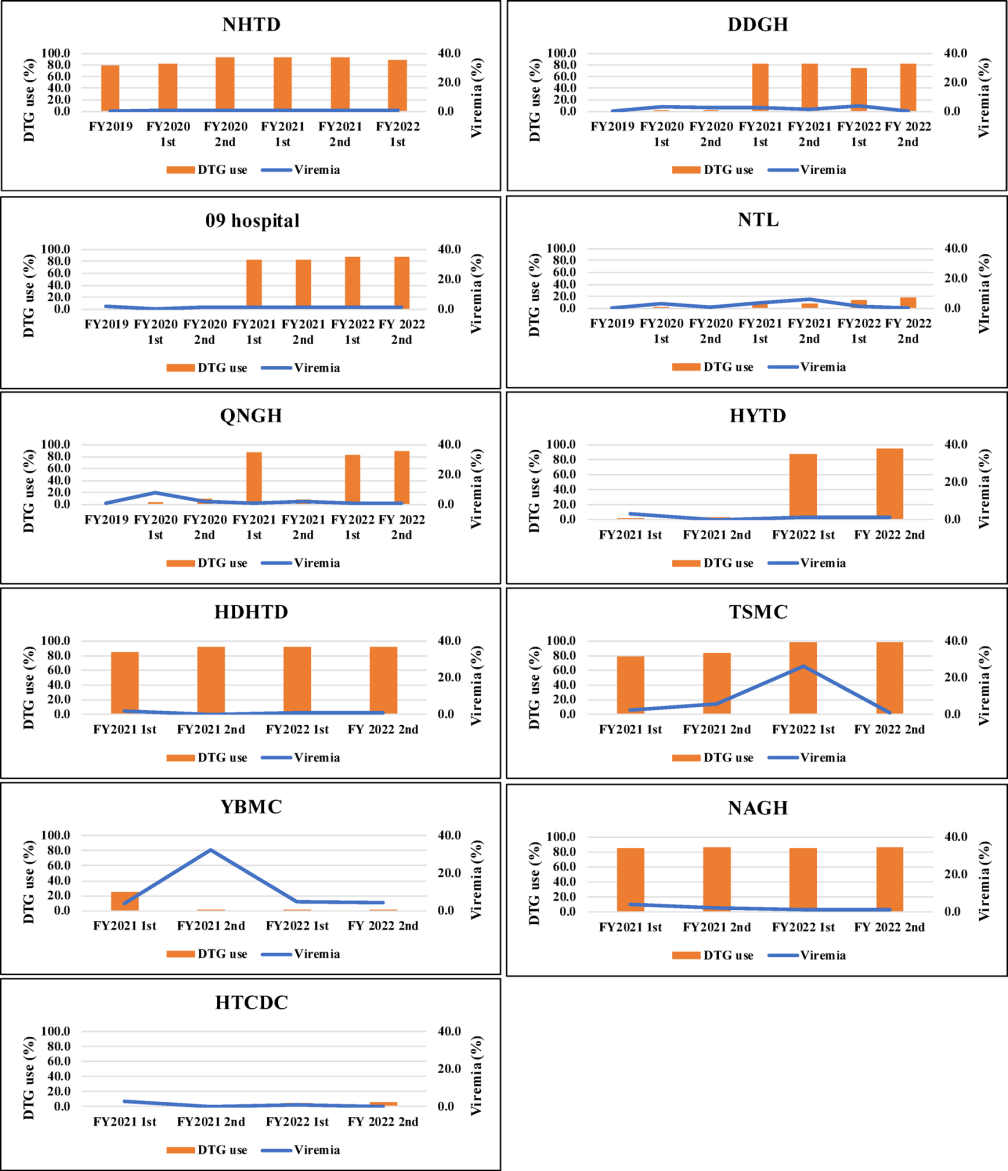
Dolutegravir (DTG) use and prevalence of viremia by study site. FY, fiscal year; NHTD, National Hospital for Tropical Diseases; DDGH, Dong Da General Hospital; NTL, Nam Tu Liem Health Center; QNGH, Quang Ninh General Hospital; HYTD, Hung Yen Hospital of Tropical Diseases; HDHTD, Hai Duong Hospital for Tropical Diseases; TSMC, Thanh Son District Medical Center; YBMC, Yen Binh District Medical Center; NAGH, Nghe An General Hospital; HTCDC, Ha Tinh Center for Disease Control and Prevention.

In total, 1,891 (84.7%) study participants received a DTG-containing regimen during the follow-up period. Of those, 1,874 (99.1%) used a combination of TDF/3TC/DTG. Of the 1,891 study participants, 41 discontinued the DTG-containing regimen in less than 12 weeks, and 34 did not have HIV-VL test results ≥ 12 weeks after DTG initiation. Among the remaining 1,816 study participants included in the analysis, the incidence of viremia after DTG initiation was 2.2/100 person-years. Figure 2 shows DTG use and prevalence of viremia according to study site. The availability of DTG-containing regimens varied by study site. In four facilities (NHTD, HDHTD, TSMC, and NAGH), most participants received a DTG-containing regimen continuously throughout the follow-up period. In five facilities (DDGH, 09 Hospital, QNGH, HYTD, and YBMC), DTG-containing regimens became available during the follow-up period. DTG use was rarely observed in two facilities (NTL and HTCDC). In general, higher-level facilities were more likely to use DTG-containing regimens than lower-level facilities. The prevalence of viremia was steadily low throughout the follow-up period, except for that in YBMC and TSMC. For YBMC, 27 of 83 (32.5%) participants under follow-up had viremia at the second study visit in FY 2021, when the rate of DTG use decreased from 24.2% to 1.2%. For TSMC, 29 of 111 participants (26.1%) had viremia at the first study visit in FY 2022, when most participants were receiving DTG-containing regimens. Although QNGH experienced a decline in DTG users (from 87.1% to 8.4%) between the two study visits in FY 2021, the change in DTG use did not result in an increase in viremia. Additionally, six participants from all facilities had HIV-DR test results before starting a DTG-containing regimen and four had any DRMs (three with DRMs to both NRTIs and NNRTIs, one with DRMs to NNRTIs only; no DRMs to PIs and INSTIs). All four participants who had already had DRMs to NRTI- or NNRTI-associated mutations before DTG initiation maintained an HIV-VL below 200 copies/mL until the end of follow-up (data not shown).

Table 3 shows the reasons for discontinuation of DTG-containing regimens by facility. In total, there were 292/1,891 (15.4%) study participants who discontinued the regimens; the most commonly reported reason was DTG stockout (80.8%), followed by drug toxicity (15.1%). Particularly in YBMC and QNGH, all participants experienced discontinuation of DTG-containing regimens owing to drug stockout. No patients discontinued the regimens owing to a lack of drug effectiveness. Among toxicities leading to discontinuation, renal dysfunction was the most common (Table 4).

**Fig. 3:**
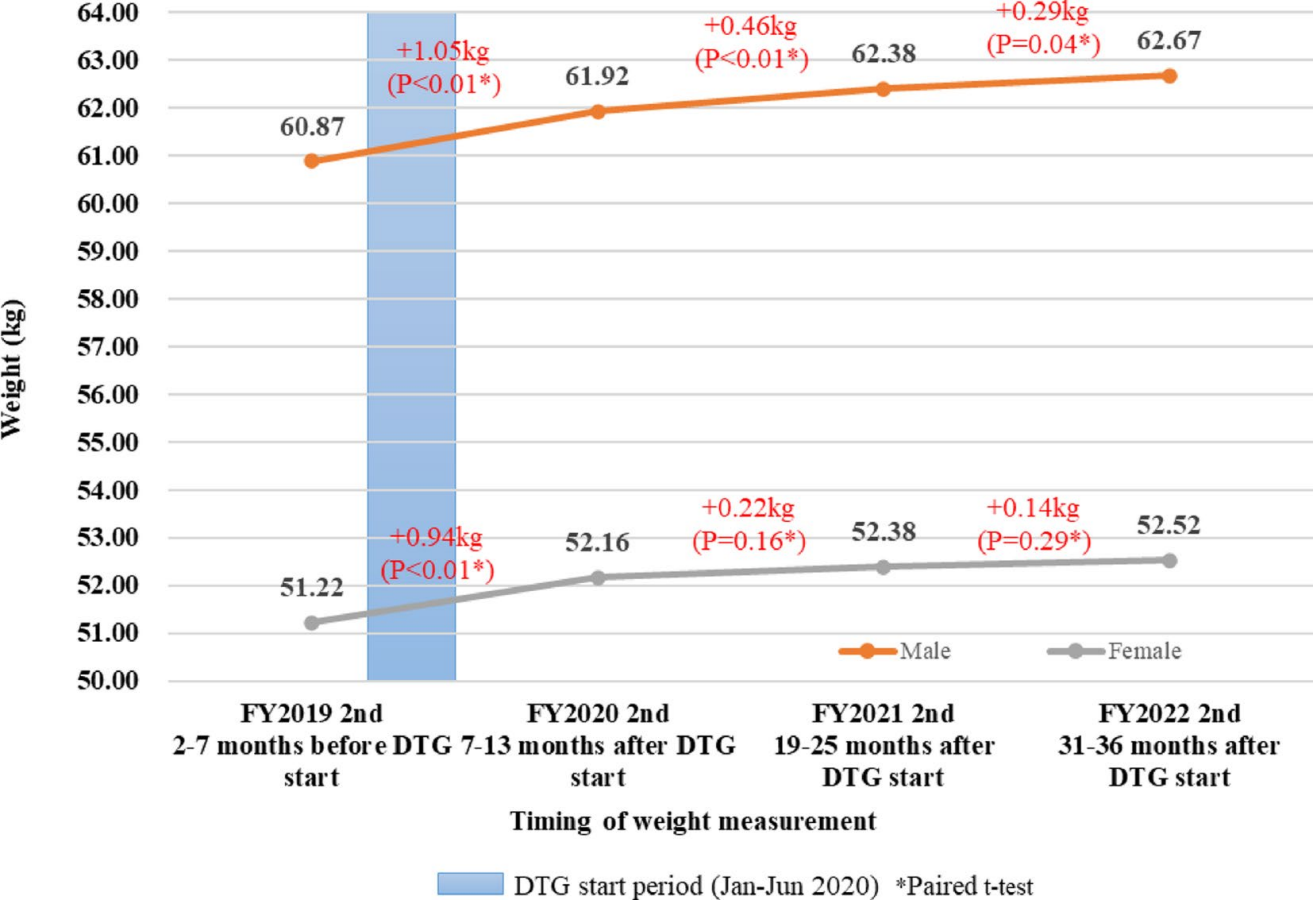
Change in average weight among dolutegravir (DTG) users at National Hospital for Tropical Diseases FY, fiscal year.

We included 571 NHTD patients to examine weight gain. All patients switched to a DTG-containing regimen between January and June 2020. The mean participant age was 42.2 years, and 54.5% were male individuals. All had a baseline HIV-VL below 200 copies/mL. Figure 3 depicts weight gain after switching to a DTG-containing regimen, according to sex. The average weight gain at the first measurement after switching to a DTG-containing regimen (7–13 months after switching) was 1.05 kg for male and 0.94 kg for female participants, respectively. The average weight gain at the first measurement after switching was greater than that for subsequent measurements, and weight gain stabilized over time. Although weight gain among male participants was statistically significant across visits (*p* < 0.04), weight gain among female participants was significant only between the first and second visits (*p* < 0.01).

**Table 3:** Reasons for discontinuation of DTG-containing regimen, by facility.

Facility	Patients who ever started DTG-containing regimen, *n*	Patients who discontinued DTG-containing regimen, *n* (%)	Reason for discontinuation of DTG-containing regimens ^a^			
			Stockout, *n* (%)	Toxicity, *n* (%)	Lack of effectiveness, *n* (%)	Other reasons, *n* (%)
All	1,891	292 (15.4)^b^	236 (80.8)	44 (15.1)	0 (0.0)	14 (4.8)^c^
NHTD	1,168	112 (9.6)	62 (55.4)	38 (33.9)	0 (0.0)	12 (10.7)
DDGH	67	1 (1.5)	0 (0.0)	1 (100.0)	0 (0.0)	0 (0.0)
09 Hospital	74	6 (8.1)	4 (66.7)	2 (33.3)	0 (0.0)	0 (0.0)
NTL	7	1 (14.3)	0 (0.0)	0 (0.0)	0 (0.0)	1 (100.0)
QNGH	90	80 (88.9)	80 (100.0)	1 (1.3)^d^	0 (0.0)	1 (1.3)^d^
HYTD	88	1 (1.1)	1 (100.0)	0 (0.0)	0 (0.0)	0 (0.0)
HDHTD	94	2 (2.1)	0 (0.0)	2 (100.0)	0 (0.0)	0 (0.0)
TSMC	126	0 (0.0)	0 (-)	0 (-)	0 (-)	0 (-)
YBMC	90	89 (98.9)	89 (100.0)	0 (0.0)	0 (0.0)	0 (0.0)
NAGH	86	0 (0.0)	0 (-)	0 (-)	0 (-)	0 (-)
HTCDC	1	0 (0.0)	0 (-)	0 (-)	0 (-)	0 (-)

^a^ Reasons for each discontinuation were counted. The median time from DTG initiation to discontinuation was 20 weeks.

^b^ Among 292 patients who discontinued DTG-containing regimens for any reason, 144 (49.3%) restarted a DTG-containing regimen with a median time interval of 44 weeks. Of those, two patients discontinued DTG more than once.

^c^ Tuberculosis (*n* = 6), pregnancy or childbirth (*n* = 3), desire to become pregnant (*n* = 3), bought medicine themselves (*n* = 1), DTG not covered by social health insurance (*n* = 1).

^d^ Two patients discontinued DTG-containing regimens for two reasons: stockout and toxicity, or stockout and other reasons.

DTG, dolutegravir; NHTD, National Hospital for Tropical Diseases; DDGH, Dong Da General Hospital; NTL, Nam Tu Liem Health Center; QNGH, Quang Ninh General Hospital; HYTD, Hung Yen Hospital of Tropical Diseases; HDHTD, Hai Duong Hospital for Tropical Diseases; TSMC, Thanh Son District Medical Center; YBMC, Yen Binh District Medical Center; NAGH, Nghe An General Hospital; HTCDC, Ha Tinh Center for Disease Control and Prevention.

**Table 4:** Toxicity leading to dolutegravir (DTG) discontinuation (multiple choice).

Toxicity (*n* = 44)	*n* (%)
Renal dysfunction	38 (86.4)
Allergic reactions	2 (4.5)
Gastrointestinal	2 (4.5)
Neuropsychiatric/hepatic/osteoarticular	0 (0.0)
Other^a^	7 (15.9)

^a^ Diabetes (*n* = 2), weight gain (*n* = 2), weight loss (*n* = 1), tiredness (*n* = 1), psoriasis (*n* = 1).

## Discussion

In this multicenter cohort study among PLHIV in Vietnam, we demonstrated successful maintenance of virologic outcomes of ART and high treatment retention amid various social and clinical changes (i.e., transition to SHI-based HIV services, COVID-19 pandemic, and introduction of DTG) that have occurred in the country in recent years. The high effectiveness and acceptable tolerability of DTG-containing regimens were also confirmed in this real-world setting, supporting the national rollout of DTG use.

HIV treatment retention was successfully maintained during the study period. Furthermore, high viral suppression (HIV-VL < 50 copies/mL) and low viremia (HIV-VL ≥ 200 copies/mL) rates were observed, as compared with reports from other countries^30–32^. Viral suppression rates were maintained above 95%, except for in FY 2021 and FY 2022, when Vietnam was severely affected by the COVID-19 pandemic and the viral suppression rates decreased to just below 95%. These recent viral suppression rates were similar to those found in our previous study conducted at NHTD from 2007 to 2015 (95.5% at 12 months)^33^ and a national survey conducted in 2020 (96.4% at 12 ± 3 months and 98.6% at ≥ 48 months after ART initiation)^2^. However, we cannot simply compare these results with the findings of our study because we only included virally suppressed patients before enrollment. Furthermore, the treatment retention rate found in this study (87.2% at 3 years after study enrollment) was similar to the finding of our previous study conducted at NHTD from 2007 to 2012. In that study, we reported a retention rate of 90.5% at 3 years after study enrollment^34^. Whereas studies in other countries reported that COVID-19 negatively impacted HIV care engagement and treatment outcomes^15,35^, the minimal decline in treatment retention and viral suppression rates found in the present study indicate that HIV treatment was resilient and viral replication was well controlled during the pandemic in Vietnam. The effective responses to COVID-19 at all HIV service levels in Vietnam, including government, provincial disease control centers, and ART clinics, as well as the close coordination among these levels, may have contributed to maintaining stable HIV treatment during the pandemic^36^.

Another possible reason for the maintenance of successful HIV treatment was the gradual integration of HIV services into the SHI scheme. The shift in the HIV financing system did not take place as originally envisioned. For example, PLHIV with low socioeconomic status are exempted from paying out-of-pocket costs for treatment whereas others, including those without an SHI card and/or Vietnamese nationality, are financed by other internal or external sources, such as the provincial People’s Committee and the Global Fund. Thus, all study participants could receive ART free of charge during the follow-up period. Moreover, the Health Insurance Law has been amended (No. 46/2014/QH13), meaning that PLHIV can receive ART at provincial-level facilities with a transfer letter even if they have registered with SHI at lower-level facilities, such as district- or commune-level ART clinics. If PLHIV wish to avoid neighborhood facilities owing to fear of stigma, they can choose the facilities at which to receive HIV services. These flexible and gradual system reforms, which are tailored to the needs of PLHIV, have likely helped them to remain engaged in HIV treatment.

Only 32 (1.4%) of 2,233 study participants had any DRMs in this study. This prevalence was lower than that found in national HIV-DR surveys conducted in 2014–2016^37^ (4.6% at ≥ 36 months after ART initiation) and in 2017–2018^38^ (3.0% and 3.4% at 12 ± 3 months and ≥ 48 months after ART initiation, respectively). Moreover, we found that 32 (46.4%) of PLHIV with treatment failure (HIV-VL > 1000 copies/mL) had any DRMs whereas previous national surveys reported higher rates, i.e., 94.8% with any DRMs at ≥ 36 months after ART initiation in 2014–2016^37^, 88.5% with any DRMs at ≥ 48 months after ART initiation in 2017–2018^38^, and 58.7% with DRMs to NNRTI at ≥ 48 months after ART initiation in 2020^2^. This may indicate that participants in this study were virologically stable before study enrollment, according to the inclusion criteria. However, even taking this into account, there would be no upward trend in the HIV-DR prevalence in Vietnam.

The favorable virological outcomes found in this study (i.e., high viral suppression rates and low drug resistance rates) may be also owing to the introduction of DTG-containing regimens. The incidence of viremia (HIV-VL ≥ 200 copies/mL) after switching to a DTG-containing regimen was low at 2.2/100 person-years, and no study participants with treatment failure had INSTI-associated DRMs. Consistent with previous studies, these real-world data confirmed the virological effectiveness of DTG-containing regimens in switch therapy and the high genetic barrier of DTG to drug resistance, even in patients with the presence of DRMs^39–41^. However, the accumulation of NRTI-associated mutations in patients switching to TDF/3TC/DTG may lead to functional DTG monotherapy and enhance the rapid emergence of DTG resistance^42,43^. Thus, continuous HIV-VL and HIV-DR monitoring is especially important for patients with a history of virological failure.

Although this study added further evidence to support the rollout of DTG-containing regimens in Vietnam, the expansion of the regimens has not progressed consistently across all regions or facility levels (Fig. 2). Among the 11 study sites, we found variations in the use of DTG-containing regimens, with the proportion of patients who have ever used DTG ranging from 1.0% to 98.4%. Additionally, of those who switched to a DTG-containing regimen during the study period, 15.4% discontinued the regimen. Most discontinuations were reported in QNGH or YBMC owing to DTG being out of stock. DTG stockouts may have occurred owing to drug supply shortages, improper management of DTG stocks, and poor communication between relevant institutions. Study participants who discontinued a DTG-containing regimen because of stockouts had to switch to an alternative regimen to continue ART, without any clinical reason. Given that QNGH maintained high viral suppression even after patients discontinued DTG-containing regimens, the association between discontinuation and the occurrence of viremia was unclear. However, at YBMC, approximately one-third of study participants under follow-up experienced viremia after DTG-containing regimens nearly became unavailable. This may suggest that stable procurement of DTG-containing regimens is an issue to be addressed so as to maintain viral suppression.

The main reason for discontinuation of DTG-containing regimens was drug stockouts; the regimens were otherwise well tolerated in this study. Although recent observational studies reported a high incidence of discontinuation of DTG-containing regimens owing to toxicity, mainly related to neuropsychiatric events^44,45^, no one reported such toxicity in this study. Regarding weight changes after switching to DTG-containing regimens, consistent with previous studies, our study found an increase in average weight, particularly immediately after starting DTG^23,24,46^. However, the weight gain observed in our study, in which the majority of patients switched from TDF/3TC/EFV to TDF/3TC/DTG, was less pronounced than reported in studies switching to tenofovir alafenamide (TAF)/3TC/DTG or ABC/3TC/DTG^46,47^. The NRTI backbone used with DTG may play a role in weight changes^48^. DTG-induced weight gain and its mechanisms are still controversial. Further researches accounting for dietary and lifestyle factors are needed. Contrarily, renal dysfunction was the second most common reason for discontinuing DTG-containing regimens in the present study. This might have reflected an increase of serum creatinine owing to inhibition of renal organic cation transporters by DTG^49^, which may have led to a mechanistic bias. However, TDF-induced nephrotoxicity may have been another possibility. It has been reported that TDF toxicity in the kidneys may be greater among Vietnamese PLHIV, who often have lower body weight than Westerners^50,51^. Although the cause of renal dysfunction was not thoroughly assessed by measuring the estimated glomerular filtration rate from cystatin C to distinguish actual nephrotoxicity in this study, the results may suggest that renal function should be closely monitored when using the combination TDF/3TC/DTG.

In addition to the challenges mentioned above, there are several issues that must be addressed. Among participants with treatment failure (*n* = 69), 16 (23.2%) had DRMs to both NRTI and NNRTI, indicating the need for a timely change to an optimal ART regimen. The 2021 Vietnamese treatment guidelines also recommend HIV-DR testing for PLHIV who are exposed to multiple ART regimens before switching to a second- or third-line regimen^52^. Nonetheless, HIV-DR testing is not offered owing to a limited number of testing institutions and a lack of financial resources to procure reagents. Moreover, drug resistance testing is not currently covered by SHI; therefore, PLHIV must pay the entire cost of testing if there is no financial support. Additionally, we found clear differences in the incidence of viremia among facility levels, with patients in lower-level hospitals having a greater probability of viremia. This result supports previous studies reporting disparities in the quality of HIV treatment and care in Vietnam according to facility level^53^. One of the challenges ahead is promoting uniformity in the quality of HIV services across all facility levels, including clinical skills for effective adherence support and ART management.

The strengths of the present study include the real-world setting and large sample size. This study also provides important information on achievements and challenges regarding HIV/AIDS treatment during the transition to SHI-based HIV services in Vietnam. However, this study also had several limitations. First, the 11 study sites were selected in consultation with Vietnam’s Ministry of Health from multiple perspectives, including region and facility level. However, the representativeness across North Vietnam of PLHIV participating in this study has not been fully validated. Second, the present study was conducted during the gradual transition to SHI-based HIV services, and we reported successful virological outcomes in recent years. However, this may have been influenced by our inclusion criterion that participants in this study were virologically stable prior to study enrollment. In addition, our study data did not allow for comparisons of virological outcomes before and after SHI implementation because the introduction of SHI did not occur in a clear-cut manner and the timing of introduction and service coverage after introduction varied from facility to facility. Similarly, DTG implementation and the COVID-19 pandemic occurred during the follow-up period of this study, with the most relevant time period varying across facilities. Therefore, our study did not assess the impact of each social/clinical change on virological outcomes. Third, although the rate of loss to follow-up in this study was low, the HIV-VL may be higher among patients lost to follow-up or those who transferred to other facilities. Fourth, the results regarding weight gain may require cautious interpretation because robust analysis could not be conducted to fully take into account the factors associated with weight gain.

In conclusion, with the gradual transition to SHI-based HIV services and the introduction of DTG-containing regimens, Vietnam has successfully maintained engagement in care and virological outcomes in recent years. These real-world data also confirm the effectiveness and tolerability of DTG-containing regimens, supporting the national rollout of DTG use.

### Ethical approval

This study was approved by both the Human Research Ethics Committee of the National Center for Global Health and Medicine (Reference: NCGM-G-003124-03) and the Bio-medical Research Ethics Committees of the National Hospital for Tropical Diseases (reference:17/HDDD-NDTU). We performed this study in accordance with Japan’s Ethical Guidelines for Medical and Health Research Involving Human Subjects, issued by the Japanese Ministry of Health, Labour and Welfare. Each participant provided written informed consent for study participation.

## Supplementary Information

Below is the link to the electronic supplementary material.

Supplementary Material 1

## Data Availability

The datasets generated during and/or analyzed during the current study are available from the corresponding author on reasonable request.
