# Supplementary material for: Virological outcomes and treatment retention in North Vietnam amidst transition to social insurance-based HIV services and dolutegravir-based regimens
Source: Sci Rep. 2025 Nov 28;15:42657. doi: 10.1038/s41598-025-26866-5 (PMC12663129; doi:10.1038/s41598-025-26866-5)
Supplement: Supplementary file 1 — Supplementary Material 1 [file 41598_2025_26866_MOESM1_ESM.pdf]

Supplementary Table 1. Facility information

Facility	Location province/city	Facility level	Enrollment	SHI coverage*	Copayment for ARVs*	Copayment for HIV-VL test*
NHTD	Hanoi	National	2020 FY (Jun – Jul 2020)	70-80%	Free (ARVs were provided by donor)	Free (VL test was provided by donor)
DDGH	Hanoi	Provincial	2019 FY (Dec 2019 – Jan 2020)	80-90%	Free (ARVs were provided by donor)	Free (SHI + co-payment was covered by donor)
09 Hospital	Hanoi	Provincial	2019 FY (Dec 2019 – Jan 2020)	50-60%	Free (ARVs were provided by National Target Program)	Free (VL test was provided by Hanoi People's committee)
NTL	Hanoi	District	2019 FY (Feb – Mar 2020)	>90%	Free (SHI + co-payment was covered by Hanoi People's committee)	Free (VL test was provided by donor)
QNGH	Quang Ninh	Provincial	2019 FY (Jan – Feb 2020)	>90%	Free (SHI + co-payment was covered by donor)	Free (VL test was provided by donor)
HYTD	Hung Yen	Provincial	2021 FY (Apr – May 2021)	>90%	Free (SHI + co-payment was covered by provincial government)	Free (SHI + co-payment was covered by donor)
HDHTD	Hai Duong	Provincial	2021 FY (Jun – Jul 2021)	>90%	Free (SHI + co-payment was covered by donor)	Free (SHI + co-payment was covered by donor)
TSMC	Phu Tho	District	2021 FY (Apr – May 2021)	>90%	Free (ARVs were provided by donor)	Free (VL test was provided by donor)

YBMC	Yen Bai	District	2021 FY (Apr – May 2021)	>90%	Free (SHI + co-payment was covered by donor)	Free (SHI + co-payment was covered by donor)
NAGH	Nghe An	Provincial	2021 FY (Jun – Jul 2021)	80-90%	Free (SHI + co-payment was covered by provincial government)	Paid (VL test was not covered by SHI. No donor support was provided)
HTCDC	Ha Tinh	Provincial	2021 FY (May – Jun 2021)	>90%	Free (SHI + co-payment was covered by Ha Tinh People's committee)	Free (SHI + co-payment was covered by donor)

SHI, Social Health Insurance; ARVs, antiretroviral drugs; HIV-VL, HIV viral load

NHTD, National Hospital for Tropical Diseases; DDGH, Dong Da General Hospital; NTL, Nam Tu Liem Health Center; QNGH, Quang Ninh General Hospital; HYTD, Hung Yen Hospital of Tropical Diseases; HDHTD, Hai Duong Hospital for Tropical Diseases; TSMC, Thanh Son District Medical Center; YBMC, Yen Binh District Medical Center; NAGH, Nghe An General Hospital; HTCDC, Ha Tinh Center for Disease Control and Prevention.

The median follow-up period for the five hospitals enrolled in FY 2019 and FY 2020 was 129 weeks whereas the median follow-up period for the six hospitals enrolled in FY 2021 was 76 weeks.

*Data was obtained at enrollment.

Supplementary Table 2. Drug resistance mutations, by drug class (n=32)

Class	DRMs	n (%)[a]
NRTIs	M184V/I	14 (43.8)
	V75M/I	11 (34.4)
	T215D/F/Y	6 (18.8)
	M41L	4 (12.5)
	D67G/N	3 (9.4)
	L210W	3 (9.4)
	E44D	3 (9.4)
	K70G/N/Q	3 (9.4)
	K65R	2 (6.3)
	T69D/N	2 (6.3)
	K219E/Q	2 (6.3)
	L74I	1 (3.1)
	S68G	1 (3.1)
NNRTIs	V106I	11 (34.4)
	K103N	8 (25.0)
	V179D/E	6 (18.8)
	K101E/N	6 (18.8)
	G190A	6 (18.8)
	P225H	6 (18.8)
	Y181C	6 (18.8)
	A98G	4 (12.5)
	V108I	3 (9.4)
	Y188L	3 (9.4)
	H221Y	2 (6.3)
	N348I	2 (6.3)
	F227L	1 (3.1)
	K101Q	1 (3.1)
	M230L	1 (3.1)
	Y318F	1 (3.1)
PIs	M46I	2 (6.3)
	I54V	1 (3.1)
	V82A	1 (3.1)

DRMs, drug resistance mutations; NRTIs, nucleoside analogue reverse transcriptase inhibitors; NNRTIs, non-nucleoside reverse transcriptase inhibitors; PIs, protease inhibitors.

[a] The percentage was calculated using the total number of participants with any DRMs as the denominator (n=32).

Supplementary Table 3. Cox proportional hazard models: factors associated with viremia (≥200)

	Univariable (n=2,205)				Multivariable (n=1,461)			
**Variables**	**HR**	**95% CI**		***p***	**HR**	**95% CI**		***p***
All								
**Facility level**								
National	1.0			<0.0001	1.0			<0.0001
Provincial	1.9	1.2	3.0		2.2	1.1	4.2	
District	10.3	6.9	15.4		6.7	3.6	12.3	
**Sex**								
Male	1.0			0.66				
Female	0.9	0.7	1.3					
**Age, years**								
<30	1.1	0.5	2.3	0.81				
30–39	0.9	0.6	1.6					
40–49	0.8	0.5	1.4					
≥50	1.0							
**Route of transmission: IDU**								
Yes	1.2	0.9	1.8	0.25				
No	1.0							
**Route of transmission: MSM**								
Yes	0.7	0.2	2.2	0.53				
No	1.0							
**Time from HIV diagnosis to registration**								
10 years	1.0			0.54				
≥10 years	0.9	0.6	1.3					
**Time from HIV diagnosis to ART initiation**								
<1 month	1.0			0.09	1.0			0.60
≥1, <6 months	0.7	0.5	1.1		1.0	0.6	1.7	
≥6, <12 months	1.1	0.6	1.9		0.6	0.2	2.0	
≥12 months	0.6	0.4	0.9		0.7	0.3	1.4	
**CD4 (/mL) before the first visit**								
<200	4.0	1.7	9.5	<0.001	2.5	1.0	6.1	0.04
200–349	2.1	1.2	3.8		1.7	1.0	3.1	
≥350	1.0				1.0			
**HIV-VL (copies/mL) at the first visit**								
<20	1.0			<0.0001	1.0			<0.01

20–199	3.2	2.2	4.8	1.9	1.0	3.6
200–999	8.3	2.6	26.4	40.5	5.2	316.2
≥1000	4.3	1.4	13.5	–	–	–

HR, hazard ratio; CI, confidence interval; IDU, injection drug use; MSM, men who have sex with men; ART, antiretroviral therapy; HIV-VL, HIV viral load.
